# Antimicrobial Activities of a Plethora of Medicinal Plant Extracts and Hydrolates against Human Pathogens and Their Potential to Reverse Antibiotic Resistance

**DOI:** 10.1155/2015/547156

**Published:** 2015-05-27

**Authors:** Dieudonné Lemuh Njimoh, Jules Clement N. Assob, Seraphine Ebenye Mokake, Dinga Jerome Nyhalah, Claude Kwe Yinda, Bertrand Sandjon

**Affiliations:** ^1^Department of Biochemistry and Molecular Biology, Faculty of Science, University of Buea, Buea, South West Region, Cameroon; ^2^Department of Medical Laboratory Sciences, Faculty of Health Sciences, University of Buea, Buea, South West Region, Cameroon; ^3^Department of Botany and Plant Physiology, Faculty of Science, University of Douala, Douala, Littoral Region, Cameroon; ^4^Phytorica Laboratory, Douala, Littoral Region, Cameroon

## Abstract

Microbial infections till date remain a scourge of humanity due to lack of vaccine against some infections, emergence of drug resistant phenotypes, and the resurgence of infections amongst others. Continuous quest for novel therapeutic approaches remains imperative. Here we (i) assessed the effects of extracts/hydrolates of some medicinal plants on pathogenic microorganisms and (ii) evaluated the inhibitory potential of the most active ones in combination with antibiotics. Extract E03 had the highest DZI (25 mm). Extracts E05 and E06 were active against all microorganisms tested. The MICs and MBCs of the methanol extracts ranged from 16.667 × 10^3^ *μ*g/mL to 2 *μ*g/mL and hydrolates from 0.028 to 333333 ppm. Extract E30 had the highest activity especially against *S. saprophyticus* (MIC of 6 ppm) and *E. coli* (MIC of 17 ppm). Combination with conventional antibiotics was shown to overcome resistance especially with E30. Analyses of the extracts revealed the presence of alkaloids, flavonoids, triterpenes, steroids, phenols, and saponins. These results justify the use of these plants in traditional medicine and the practice of supplementing decoctions/concoctions with conventional antibiotics. *Nauclea pobeguinii (E30)*, the most active and synergistic of all these extracts, and some hydrolates with antimicrobial activity need further exploration for the development of novel antimicrobials.

## 1. Introduction

The folkloristic concepts of a wide range of medicinal plants have been proven scientifically and the latter has led to the development of drug regimens to fight various infectious diseases impeding human life and activity. While plants remain the natural and undisputable reservoir of anti-infectious agents, the quest for scientific validation and development of new drugs or therapeutic combinations from yet unexplored plants used in traditional pharmacopoeia remains very imperative due to resurgent problems of resistance, affordability, and efficacy. Antimicrobial, especially antibiotic drug resistance is a challenge to public health despite the existence of a variety of antibiotics [1]. Caused mostly by the unregulated use of antimicrobials and poor hygienic conditions amongst others, it severely affects humans in every aspect of life [2]. Though immunization through vaccines has resulted in the control and eradication of some microbial infections [3] a vast majority rely solely on chemotherapy, which is constantly hampered by the emergence of resistant phenotypes. The increasing prevalence of these resistant phenotypes especially those with multiple resistances is responsible for most of the difficulties encountered in treating these diseases. Bacterial and fungal infections account for the greatest part of infections found in health services. While bacterial infection alone accounts for about 90% of these infections, fungal infections are on the rise in developing countries because of opportunities of coinfection like with HIV and other medical manipulations [4].

Plant extracts and essential oils have been widely explored for their therapeutic activities against most microbial infections [5]. But virtually very little has been done on the antimicrobial profile of hydrolates (obtained from the steam distillation of medicinal and aromatic plants). About 80% of the world's population relies on plant derived medicines for their primary health and 3.5 billion people in the developing world depend on the exploitation of medicinal plants and herbal products around them for their healthcare needs [6]. Though this approach of traditional medicine has not, in most cases, been scientifically validated for their safety and efficacy, various reasons account for its continuous large scale application, irrespective of the presence of conventional medicine. Some of these herbal products, though curative, have been associated with severe diseases and organ failures [7]. Thus their evaluation for safety and efficacy remains an important challenge. This study was aimed at demonstrating the antimicrobial activity of a number of crude extracts and hydrolates from medicinal plants used in folk medicine (Table 1) and to evaluate their potentials to act in synergy with conventional antibiotics to which microorganisms have developed resistance. Using concoctions/decoctions in combination with antibiotics to which microorganisms have developed resistance to treat microbial infections is increasingly becoming popular and more effective in traditional pharmacopoeia. Hydrolates have been used in traditional medicine to treat various ailments and some have been associated with mosquito larvicidal activity [8, 9]. Their antimicrobial activity has not been well explored despite the fact that they have been reported to contain many bioactive hydrophilic compounds amongst which are phenols, alcohols, and terpenes which can make potential antimicrobial candidates.

## 2. Materials and Methods

### 2.1. Collection and Identification of Plant Materials

Medicinal plants and plant parts (Table 1) used in this study were collected between 2007 and 2011 from the Bassa land (between the centre and the Littoral Region) of Cameroon. Specimens were identified at the Cameroon National Herbarium in Yaoundé, Cameroon.

### 2.2. Preparation of Crude Extracts and Hydrolates

Fresh plant materials were air-dried at room temperature and crushed into powder and extraction was done by macerating the powder in 6 litres of hexane for two days. The filtrate was evaporated to dryness under reduced pressure using a rotary vacuum evaporator. The concentrate was macerated in 6 litres of methanol (MeOH). The concentrated crude extracts obtained from the latter after filtration/evaporation were stored at 4°C. Hydrolates were obtained by separating the aqueous water from essential oils after steam distillation in the course of extracting essential oils.

### 2.3. Processing of Test Substances and Cells

Each extract was prepared by dissolving 250 mg in 5 mL of 10% (v/v) aqueous Dimethyl Sulfoxide (DMSO) to give an assay concentration of 50 mg/mL. Gentamycin and Nystatin (both from Sigma, USA) served as positive controls and were used at a concentration of 0.2 mg/mL, respectively, for bacteria and fungi. A stock solution of these controls was prepared by dissolving 10 mg in 5 mL of 10% (v/v) DMSO. Hydrolates were not resuspended in DMSO because of their watery nature. Clinical isolates of six pathogenic bacteria (*Escherichia coli, Klebsiella pneumoniae, Salmonella typhi, Staphylococcus aureus, Staphylococcus epidermidis, *and* Staphylococcus saprophyticus*) and two fungal species (*Trichophyton rubrum* and* Candida albicans*), obtained using selective media following standard protocols [10, 11] from the Regional Hospital Annex in Buea in the Southwest Region of Cameroon, were used. Isolates were identified by their ability to grow on selective media, by gram reaction, by their morphology and by various distinct biochemical reactions [12].

### 2.4. Susceptibility Tests and Determination of MIC and MBC

The hole-plate diffusion and broth microdilution methods were used as described [13] to assess the antimicrobial susceptibility of extracts and hydrolates, respectively, by measuring the diameter of zone of inhibition and determining the minimal inhibitory/bactericidal concentrations (MICs and MBCs). Briefly, the hole-plate diffusion method consisted of performing a uniform spread of bacteria suspension (about 100 *μ*L, corresponding to Mc Farland standard 2) on a Mueller-Hinton agar plate followed by the creation of wells of 6 mm diameter on labeled positions of the bacteria lawn and filling respective wells with 120 *μ*L (corresponding to 50 mg/mL of prepared extracts) of test samples. Only extracts E01–E14 (concentrated extracts; Table 2) were tested by this method. Positive control wells contained gentamycin and nystatin, respectively, for bacterial and fungal species while 10% DMSO served as the negative control in both cases. Plates were incubated at 37°C for 24 hours and zones of inhibition measured. Extracts that showed very significant activities with the hole-plate were further assayed together with the hydrolates for the determination of their MICs and MBCs using 96-well microtitre plates as previously described [14]. Fifty microliters of 5% glucose and 1% phenol red-supplemented nutrient broth were pipetted into duplicate wells in each microtitre plate followed by 50 *μ*L of 16.667 mg/mL DMSO-diluted sample or 333333 ppm hydrolate prepared as described above uniquely into the first well of the test wells. The wells were then diluted serially by transferring 50 *μ*L from the first well to the next until the eleventh. 50 *μ*L was discarded from the eleventh well. Positive control wells were similarly treated. Negative control wells (12th well) had no sample. 50 *μ*L of the test organism suspension was then added to each well and plates were incubated at 37°C for 24 hours and visual observation of growth was based on the colour change of the phenol red indicator from red to yellow depicting acid waste produced by the growth of the microorganism. The concentration of the well containing the lowest sample concentration that prevented visible growth or change in colour was considered the MIC. To further ascertain the MIC and to determine the MBC, 10 *μ*L of the content of the well with the MIC and the two preceding ones were used to inoculate solid agar plates. After 24 hours incubation at 37°C the well with the least growth was considered to be the MBC well and the corresponding concentration the MBC.

Susceptibility test was also performed as described [15] to identify the bacteria species resistant to selected 4 commonly used commercial antibiotics (amoxicillin, ampicillin, ceftriaxone, and norfloxacin) in order to assess the potential of the most active extracts to reverse the antibiotic resistance. A 0.5 Mc Farland standard (1.5–2 × 10^8^ CFU/mL) of each bacterium was prepared and used to inoculate the surface of Mueller-Hinton agar plate. Commercial sensitivity discs imbibed with various antibiotics were placed on the surface of the agar and after allowing the plate to stand at room temperature for 30 minutes (prediffusion time) the plate was then incubated at 37°C for 24 hours. Inhibition zones were measured. In order to determine if a bacteria culture was resistant, intermediate, or sensitive to an antibiotic, its zone of inhibition was compared with the zone diameter interpretative chart of the National Committee for Clinical Laboratory Standards [16]. To determine the MICs of these commercial antibiotics (amoxicillin, ampicillin, ceftriaxone, and norfloxacin; all acquired from the pharmacy), 20 *μ*g/mL of each was prepared in 10% DMSO and their MICs were determined in a similar manner as described above.

### 2.5. Determination of the Potential of the Most Active Extracts to Reverse Antibiotic Resistance

The MICs of the most active extracts were prepared alongside those of the conventional antibiotics. Bacteria were then cultured in wells of 96-well microtitre plates in the presence of extract, extract plus antibiotic, and antibiotic alone. Broth microdilution was then performed as described above and after incubating the plates at 37°C for 24 hours the MICs of the extract/antibiotic, and antibiotic and extract singly were then determined. Synergy between antibiotic and extract or reversal of resistance was said to occur if the MIC of the combination was less than that of the drug and the extract separately. Antagonism occurs when the combination of the MICs of extract and drug failed to inhibit the growth of the bacteria [17, 18].

### 2.6. Phytochemical Studies

Various extracts were also analyzed by various phytochemical tests and by thin layer chromatography (TLC) in order to evaluate their chemical composition. The phytochemical screening was done essentially as described [19, 20]. Normal phase silica gel GF precoated TLC plates were used to analyze the compounds present in the active crude plant extracts as previously described [21].

### 2.7. Statistical Analysis

Diameter zones of inhibition of extracts are reported as mean ± standard deviation.

## 3. Results

### 3.1. Antimicrobial Activity of Crude Extracts

A total of 14 crude extracts, E01 to E14 (Table 2), were screened against 6 bacteria and one fungal test organisms by the hole-diffusion method. The absolute values of the diameter zones of inhibition (DZI) varied from 0 to 25 mm (Table 3). The methanol extract of* Mitracarpus scaber* (E02) and especially those of* Albizia lebbeck* (E05) and* Baillonella toxisperma* (E06) were active against all the clinical isolates of microbial pathogens tested. The highest DZI was obtained with* Aframomum danielli* (E03) (25 mm) against* E. coli* and it was also active against* S. saprophyticus* (11 mm) with lesser or no activity against the other strains.* Escherichia coli* stood out as the most susceptible strain, inhibited by all 14 extracts. Extracts E11 and E14 (resp., ethanol extract of* Aucoumea klaineana* resin and hexane extract of* Clausena anisata* leaf) were only active against* E. coli*. Extracts E07 (*Fagara leprieuri*) and E10 (*Xylopia aethiopica*) were also only active against* E. coli* and very less so against* C. albicans* while extract E09 (methanol fruit extract of* Tetrapleura tetraptera*) was less active against all the strains including* E. coli*. The other extracts were inactive, less active or moderately active (Table 3).

### 3.2. Minimal Inhibitory/Bactericidal Concentrations (MICs/MBCs) of Active Extracts

**Figure 1:**
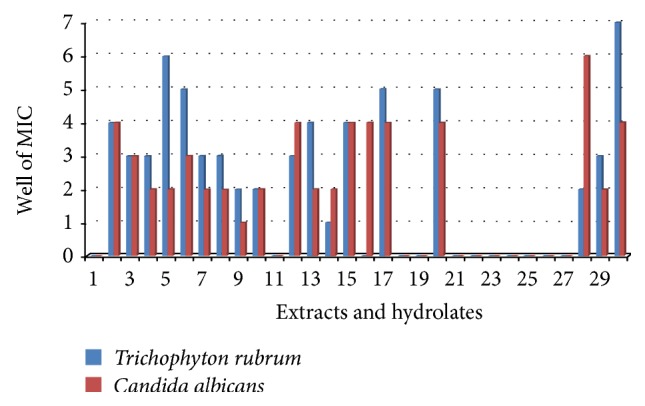
MICs of extracts against* Tricophyton rubrum* and* Candida albicans*. 1–30 = E01–E30. E02, E03, and E15 showed similar trends. E30 was the most active against* T. rubrum*. MICs: 1 = 1667 *μ*g/mL, 2 = 556 *μ*g/mL, 3 = 185 *μ*g/mL, 4 = 62 *μ*g/mL, 5 = 21 *μ*g/mL, 6 = 7 *μ*g/mL, 7 = 2 *μ*g/mL, and - = no result.

**Figure 2:**
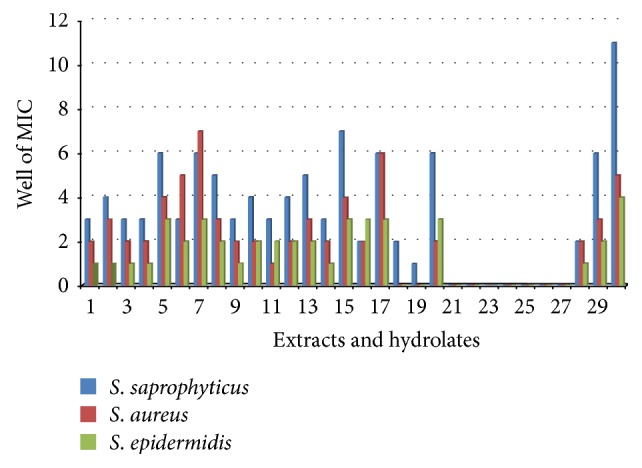
MICs of extracts and hydrolates against gram positive bacteria:* Staphylococcus saprophyticus, Staphylococcus aureus, *and* staphylococcus epidermidis*. E30 was the most active extract against all the gram positive bacteria. 1–30 = E01–E30. MICs: 1 = 1667 *μ*g/mL, 2 = 556 *μ*g/mL, 3 = 185 *μ*g/mL, 4 = 62 *μ*g/mL, 5 = 21 *μ*g/mL, 6 = 7 *μ*g/mL, 7 = 2 *μ*g/mL, and - = no result.

**Figure 3:**
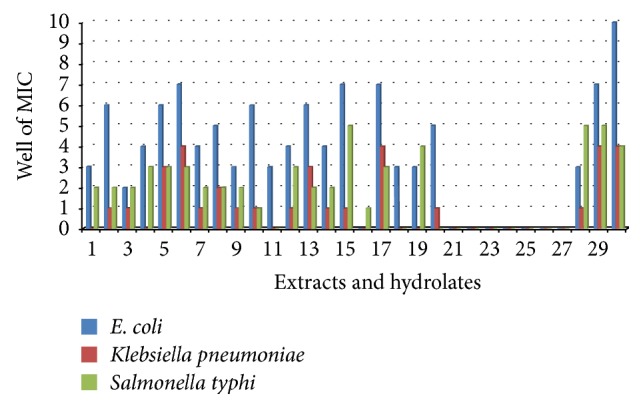
MICs of extracts and hydrolates against gram negative bacteria species:* Escherichia coli, Klebsiella pneumoniae, and Salmonella typhi.* 1–30 = E01–E30.

The minimal inhibitory concentration (MIC) of 17 extracts and 13 hydrolates were determined for various test organisms including the clinical fungal isolate* Tricophyton rubrum*. The MICs ranged from 2 *μ*g/mL to 16.667 mg/mL and 333333 ppm to 6 ppm, respectively, for crude extracts and hydrolates and liquid extracts. The MICs and MBCs of the extracts and hydrolates/liquid extracts are summarized in Tables 4 and 5, respectively. MIC < 100 *μ*g/mL was considered significant. Extracts E02, E03, and E15 had similar susceptibility trends with both fungal species while E01, E11, E18-E19, and E21–E27 did not show any activity against either of the two fungi (Tables 4 and 5). Though both fungi showed similar trends,* T. rubrum* was not susceptible to extract E16 while* C. albicans* was (MIC = 12346 ppm) (Figure 1). Also for almost all the active extracts, the MICs for* T. rubrum* were relatively lower. Extract E30 had an overall lowest MIC against* T. rubrum* (MIC = 457 ppm). Extract E30 had the lowest MIC (most active) against gram positive bacteria.* Staphylococcus saprophyticus* was the most susceptible gram positive bacteria compared to* S. aureus* and* S. epidermidis* (Figure 2).* Escherichia coli* was the most susceptible of the gram negative bacteria (Figure 3). Though* K. pneumoniae* and* S. typhi* had similar susceptibility vis-à-vis a few extracts (E05, E08, E10, E13, and E30),* S. typhi* was more susceptible than* K. pneumoniae* (Figure 3). Extracts E29 and E30 were the most active extracts against gram negative bacteria while extracts E11 and E18 were only active against* E. coli.* Extract E16 was the only extract that did not show activity against* E. coli* (the most susceptible specie) as well as against* K. pneumoniae* (Figure 3). Overall, extract E10 had the lowest MIC with gram negative bacteria species. Some MIC wells which showed growth inhibition also showed bacterial growth on solid nutrient agar. No MBC was recorded for such wells within the concentration ranges tested showing that the active samples were only bacteriostatic.

### 3.3. Antibiotic Potentiation

#### 3.3.1. Susceptibility Test

Susceptibility test was conducted with 4 antibiotics (ceftriaxone (CRO), amoxicillin (AMX), ampicillin (AMP), and norfloxacin (NOR)) against the above 6 bacteria species. The DZI of antibiotic discs against bacteria species was interpreted as resistant (R), intermediate (I), or sensitive (S) using the zone diameter interpretative chart of the NCCLS (2003) (Table 6). Staphylococcus* epidermidis*,* S. aureus*, and* S. typhi* were resistant to CRO, AMX, and AMP while* K. pneumoniae* was resistant to both AMP and AMX.* Escherichia coli* and* S. saprophyticus* were resistant to NOR and AMX, respectively.

#### 3.3.2. MIC of Antibiotics

Twenty milligrams of each of the antibiotics were used to evaluate their MICs against the bacteria to which they are resistant. The MIC ranged from 2.2 *μ*g/mL to 1.13 × 10^−4^ *μ*g/mL (Table 7).

#### 3.3.3. Extract-Antibiotic Synergism

Four best active extracts (E05, E06, E17, and E30; Tables 4 and 5) were selected to evaluate their potential to reverse antibiotic resistance. Synergy between antibiotic and extract or reversal of resistance was said to occur when the MIC of the combination was less than the MIC of the drug and the extract separately. Antagonism was said to occur when the combination of the MICs of extract and drug failed to inhibit the growth of the bacteria (Table 8). Almost all of the extracts and hydrolates chosen acted in synergy with at least one antibiotic against at least one of the test organisms. With* Staphylococcus aureus*, extract E30 (methanol root extract of* Nauclea pobeguinii*) acted in synergy with CRO, AMP, and AMX while extract E05 (methanol bark extract of* Albizia lebbeck*) and E17 (seed hydrolate of* Aframomum sulcatum*) each acted in synergy with AMP and CRO, respectively. Extract E06 (methanol bark extract of* Baillonella toxisperma*) had synergistic effect with CRO and AMP against* S. epidermidis*. With* S. saprophyticus*, all the extracts had antagonistic effect with AMX while with* Escherichia coli,* E17 and E30 acted in synergy with NOR. Also, when the extracts were used against* Klebsiella pneumoniae* in combination with AMP and AMX, E05, E06, and E17 showed no effect with AMP and were antagonistic with AMX, while E30 was synergistic with both AMP and AMX. Furthermore, when these extracts were tested in combination with CRO, AMP, and AMX against S*. epidermidis*, E06 reversed the resistance of CRO and AMP while E30 acted in synergy with AMP. When the same extracts were used in combination with CRO, AMP, and AMX against* S. typhi,* E17 and E30 acted in synergy with AMX and CRO, respectively. With the two gram positive test organisms* S. aureus* and* S. epidermidis*, E30 acted in synergy with AMP.* Nauclea pobeguinii* (E30) acted in synergy with at least one of the antibiotics in all the test organisms except* Staphylococcus saprophyticus.* Resistance due to* S. saprophyticus* could not be reversed by any of the active extract or hydrolate. Methanol root extract* of Nauclea pobeguinii* (E30), the most active extract against bacteria and fungi, had synergistic effect with at least one of the resistant antibiotics against various strains.

### 3.4. Phytochemical Analysis and TLC

The phytochemical analysis of various selected extracts using phytochemistry tests showed that the methanol extract of* Albizia lebbeck* bark (E05), hydrolate of* Aframomum sulcatum* seeds (E17), methanol extract* of Nauclea pobeguinii* roots (E30), and the methanol extract of* Baillonella toxisperma* bark (E06) all contained alkaloids and E05, E06, and E17 in addition contained triterpenes, flavonoids, and glycosides. E05 and E06 also contained phenols and saponins. E17 also contained steroids while E30 only contained alkaloids and steroids (Table 9). Thin layer chromatography (TLC) profiling showed the hydrolate of* Aframomum sulcatum* seeds (E17) to have the highest number of constituents. E17 also had the highest number of lignins after spraying the TLC plate with sulfuric acid. Using Dragendorff's reagent to test for the presence of alkaloids, it was confirmed that all the 4 extracts contained alkaloids. Finally, viewing the thin layer chromatogram under UV light yielded blue fluorescence suggesting the presence of lignans, isoflavones, and flavonoids and dark yellow spots indicating the presence of flavonoids.

## 4. Discussion 

The lack of vaccines for some microbial infections and the emergence and widespread occurrence of drug resistant phenotypes especially multidrug resistance have made fungal and bacterial diseases still a major health concern. Plants have been a cornerstone in traditional folk medicine to treat microbial infections and they also constitute sources of conventional antimicrobials. Bioassay guided fractionation and isolation of pure compounds with antimicrobial activities at times lead to a reduced or loss in activity probably because some of the compounds act in synergy. This has led to the increasing need to standardize and prioritize plant extracts as a novel approach in treating microbial infections. This study was aimed at demonstrating the antimicrobial activity of a number of crude extracts and hydrolates from medicinal plants used in folk medicine and to evaluate their potentials to act in synergy with conventional antibiotics against microorganisms which have developed resistance.

Fourteen crude plant extracts were screened by the hole-diffusion method giving a diameter of zone of inhibition (DZI) ranging from 0 to 25 mm. The methanol extract of* Xylopia aethiopica* fruit (E01) presented an important activity against* E. coli* (DZI > 10 mm) and a weak activity against* S. typhi* (1 < DZI < 4 mm) according to standard classification [22]. A similar result was obtained in Nigeria [23].* Mitracarpus scaber* whole plant MeOH extract (E02) showed moderate activity against* C. albicans* and the six bacteria strains tested. This is in line with studies conducted in Mali [24] where the methanol extract had activity against* C. albicans, E.coli, S. aureus, and K. pneumoniae.* Methanol extract of* Aframomum danielli* seeds (E03) had the highest activity (DZI = 25) against* E. coli* and this is similar to the result previously obtained [25]. The methanol extracts of* Albizia lebbeck* bark (E05) and* Baillonella toxisperma* bark (E06) were the most active as they showed activity against the 7 microorganisms tested with DZI ranging between moderate and very active. Similar values were obtained with the seed and leaf extracts of E05 [26], but no study to the best of our knowledge has demonstrated the antimicrobial property of its bark extracts. Also, no antimicrobial activity of E06 has so far been reported. The methanol stem bark extract of* Kigelia africana* (E07) and methanol fruit extract* of Tetrapleura tetraptera* (E08) showed weak to moderate activity against all the organisms tested by this method, in concordance with the results obtained previously with E07 [27]. The result obtained for E07 confirms its usage in the Cameroonian folk medicine in treating varied diseases including HIV, opportunistic infections like diarrhoea [28], genital itches, impotence, piles [29], malaria, and diabetes [27]. Methanol extract of* Tetrapleura tetraptera* fruits (E09),* Xylopia aethiopica* stem bark (E10),* Aucoumea klaineana* resin (E11),* Pamplemousse pepin* seed (E12), and* Myrianthus arboreus* root (E13) and the hexane extract of* Clausena anisata* (E14) showed no activity except against* E. coli*, which was the most susceptible organism against which all extracts showed activity.

The 14 crude extracts together with three liquid extracts and 13 hydrolates were tested by the broth microdilution assay to assess their minimal inhibitory concentrations against 8 test organisms, 6 clinical bacteria species, and 2 clinical fungal species (Tables 4 and 5). Fourteen crude extracts in solid form (E01–E014) were already tested by the hole-diffusion method while the 3 liquid extracts (methanol extract of* Scleria striatinus* roots (E15), hexane extract of* Scleria striatinus* roots (E20), and methanol extract of* Nauclea pobeguinii* roots (E30)) and the 13 hydrolates (E16–E19 and E21–E29, Table 2) were not tested by the hole-diffusion method. Some of the extracts that did not show activity by the hole-diffusion method (E09, E10, E12, E13, and E14) demonstrated activity by the broth microdilution method. Although this is quite contradictory, it demonstrates that many factors influence these methods [30].

The hydrolates of* Aframomum melegueta* seeds (E18),* Fagara zanthoxyloides* fruits (E19),* Piper nigrum* fruits (E21),* Pentadiplandra brazzeana* roots (E22),* Echinops giganteus* roots (E23),* Cupressus leylandii* leaves (E24),* Eugenia caryophyllus* fruits (E25),* Cymbopogon winterianus* leaves (E26), and* Aframomum kayserianum* seeds (E27) showed no activity (E21–E27) or was only moderately active (E18, E19) against only one to three test organisms. Seed hydrolate of* Aframomum sulcatum* (E17), fruit hydrolate of* Piper guineense* (E28) and* Piper capense* (E29), and root methanol extract of* Nauclea pobeguinii* (E30) had activity with all the test organisms. Though the seed of E17 has been used to treat male fertility and some bacteria related infections in Cameroon [31] antimicrobial activity has not yet been reported. The ethanol and water leaf extracts of* Piper guineense* (E28) have previously been shown to be active against* E. coli* and* S. aureus* while the methanol [32] and hexane fruit and root extracts of* Piper capense* (E29) have been shown to demonstrate activity against* E. coli*,* K. pneumoniae,* and* C. albicans* [33]. Hydrolates E16, E17, E28, and E29 were relatively active on almost all the microorganisms and this is the first time antimicrobial activity of the hydrolates of these plants species is being demonstrated. The leaves of* Nauclea pobeguinii* are used for the treatment of malaria in the DR Congo [34] while the stem bark is used for the prevention of threatened abortion in Upper Nyong Valley in Cameroon [35]. Its antiplasmodial activity has been reported before [34]. However, no antimicrobial activity has been reported. In this study the methanol root extract of* Nauclea pobeguinii* showed the highest activity against* S. saprophyticus* followed by* S. epidermidis*. It was the most active extract having the lowest MIC and showing activity against all the test organisms ranging from 6 to 12346 ppm.

Extracts of* Albizia lebbeck* (E05),* Baillonella toxisperma* (E06), and* Nauclea pobeguinii* (E30), and the hydrolate of* Aframomum sulcatum* (E17), were chosen on the basis of activity to investigate their potential to act in synergy with an antibiotic to which microorganisms have developed resistance. The resistance status of the clinical bacteria species revealed that all the bacteria species were resistant to at least one of the four antibiotics (ceftriaxone (CRO), amoxicillin (AMX), ampicillin (AMP), and norfloxacin (NOR)) used in this study. Staphylococcus* epidermidis*,* S. aureus*, and* S. typhi* were resistant to CRO, AMX, and AMP while* K. pneumoniae* was resistant to both AMP and AMX.* Escherichia coli* and* S. saprophyticus* were resistant to NOR and AMX, respectively. The resistance of these antibiotics was expected since they are relatively cheap and therefore easily accessible to the population who tend to abuse their use leading to resistance as earlier described [36]. E05 acted in synergy only with AMP against* S. aureus.* Of the 4 extracts, it showed the least tendency to potentiate resistant commercial antibiotics. E30 was synergistic with at least one of the antibiotics for all the test organisms except* S. saprophyticus*. A similar result has been reported for many different plants [37, 38]. This shows that such extracts may contain compounds that have different modes of action against pathogenic bacteria so that when in combination with a resistant antibiotic, they reinforce the action of the antibiotic thereby reducing the MIC. E30 was thus the best extract that demonstrated a potentiating ability. Results of the present study suggest that the concurrent administration of extracts with any of the conventional antibiotics may not necessarily elicit antagonisms as earlier thought [39]. In orthodox medicine, a plant may be subjected to several chemical processes before its active ingredient is extracted, refined, and made ready for consumption, while in traditional medicine, a plant is simply eaten raw, cooked, or infused in water or native wine, or prepared as food [40]. Hence it may be important to standardize the active extracts and administer them singly or together with conventional antibiotics to which resistance has been developed at known dosage. In traditional medicine, plants have a long history of usage in remedying many infectious diseases. This implies that the safety of concoctions from these plants is to a certain extent assured. Hence acute toxicity is essential but not very critical for most of these plants that have a long history of medicinal usage in folk medicine.

The presence of alkaloids, triterpenes, sterols, tannins, and glycosides in the three crude extracts and the floral water could account for the important antimicrobial activity exhibited by these plants against all the tested microorganisms [41]. Phytochemical analysis showed the methanol extract of the roots of* Nauclea pobeguinii* to contain only alkaloids and steroids and in high concentrations. This may also account for its very high activity. The thin layer chromatography (TLC) profile clearly shows that the hydrolate of the seeds of* Aframomum sulcatum (E17)* had a greater number of chemical components. This could be explained from the stand point of its preparation procedure. Floral water is obtained by steam distillation of plant part of interest. Essential oils in the plants and other volatile substances rise up with the steam. The steam is captured in the distillation apparatus and cooled down. The cooled condensate contains extracted water and essential oil, with the latter floating on top, and it can be skimmed off as hydrolate. Therefore hydrolate contains water soluble materials which have been extracted from the plant, including the plant juice itself, and that explains its high chemical diversity. Hydrolates have mostly been known for their role in cosmetic and not much as curative agents.

## 5. Conclusion

This study has led to the delineation of a number of plant extracts and especially hydrolates with broad spectrum antimicrobial activities justifying their usage in folk medicine to treat various ailments of microbial origin. These active extracts have chemical components like alkaloids, flavonoids, tannins, glycosides, triterpenes, sterols, and lignans that may be responsible for their powerful antimicrobial property. The hydrolate of* Aframomum sulcatum* and the crude extracts of* Albizia lebbeck*,* Baillonella toxisperma* and especially* Nauclea pobeguinii* extract have demonstrated synergism with some conventional antibiotics to which some microorganisms have developed resistance also justifying the current trend in traditional pharmacopoeia of supplementing decoctions/concoctions with antibiotics irrespective of whether or not resistance has been developed against the antibiotic in question. These three extracts (especially* Nauclea pobeguinii*, the most active and synergistic) and hydrolate are worthy of further investigation in view of isolating pure compounds for antimicrobial drug development.

## Figures and Tables

**Table 1:** Selected ethnomedicinal plants (from the Bassa land, Cameroon) used in the study and their traditional usage.

s/n	Name of plants/families	Traditional usage
	*Aframomum danielli* (Zingiberaceae)	*Aframomum danielli* seed extracts are used against inflammations and helminthic diseases, as preservatives [42].

	*Aframomum* *kayserianum * (Zingiberaceae)	Used to treat various ailments and cancer [43].

	*Aframomum melegueta* (Zingiberaceae)	*Aframomum melegueta* seeds are used to treat various ailments amongst others measles, leprosy, helminthic diseases, dysmenorrhea, bronchopulmonary disorders, female sterility, migraines, and sexual asthenia [43, 44].

	*Aframomum sulcatum* (Zingiberaceae)	*Aframomum sulcatum* seeds are used to treat male infertility, fever, and worm diseases [31].

	Albizia lebbeck (Mimosaceae)	Used to cure blood diseases, bronchitis, asthma, snakebites, hemorrhoid, itching [45].

	*Aucoumea klaineana* (Burseraceae)	Tree bark used against cough, chest pain, abscess, dysentery; resin used in skin, hair, and nail care [46, 47].

	*Baillonella toxisperma* (Sapotaceae)	Bark used to treat syphilis, kidney pain, anaemia, female infertility, sexual impotence, intestinal worms, diabetes, back aches, and itches and as purgative [48, 49].

	*Canarium schweinfurthii* (Burseraceae)	Fruits, stems, and barks used to treat cough, veneral diseases, and exudates; used as antioxidants [50].

	*Cinnamomum zeylanicum* (Lauraceae)	Used in cancer and diabetes [51].

	*Clausena anisata* (Rutaceae)	Used against gut disturbances, oral candidiasis, and fungal infections of the skin, in epilepsy and as an anticonvulsant, in high blood pressure, as antimalarial medicine, and to treat various microbial and viral infections [52, 53].

	*Cupressus leylandii* (Cupressaceae)	Used for screening or hedging.

	*Cymbopogon winterianus* (Poaceae)	Used as antibacterial, antifungal, antiyeast, insecticidal, and insect repellent agents [54].

	*Echinops giganteus* (Asteraceae)	Used to treat cancer [55].

	*Eugenia caryophyllus* (Myrtaceae)	Used to treat a broad range of diseases including abscess, tonsillitis, tuberculosis, influenza, hepatitis, cholera, malaria, and difficult childbirth [56].

	*Fagara leprieuri* (Rutaceae)	Use as anticancer agent.

	*Fagara xanthoxyloides* (Rutaceae)	Used to treat inflammations, abdominal pains, ulcers, toothache, and fever [57].

	*Kigelia africana* (Bignoniaceae)	Bark and fruits used to treat HIV and various opportunistic diseases, diarrhoea, impotence, hemorrhoid, malaria, diabetes, and genital itches [27, 28].

	*Mitracarpus scaber* (Rubiaceae)	Used to treat headache, amenorrhoea, leprosy, and skin and liver diseases [24].

	*Myrianthus arboreus* (Moraceae)	Used to treat jaundice, dysentery, diarrhoea, vomiting, fever, heart disorders, and dysmenorrhoea [58].

	*Nauclea pobeguinii* (Rubiaceae)	Used to treat malaria and to prevent miscarriages.

	*Pamplemousse pepin* (Rutaceae)	Used as natural antibiotics and antifungal agents.

	*Pentadiplandra brazzeana* (Pentadiplandraceae)	Used as antiseptic in treatment of wounds, analgesic in treatment of dental caries and rheumatism, and as aphrodisiac [59].

	*Piper capense* (Piperaceae)	*P. capense* is used to treat veneral diseases, paralysis, infertility, and heart and kidney diseases and as sexual stimulant amongst others [60].

	*Piper guineense* (Piperaceae)	*P. guineense* leaves are aperitifs, carminative, and eupeptic and are also used to treat cough, bronchitis, intestinal disorders, and rheumatism [61].

	*Piper nigrum* (Piperaceae)	*Piper nigrum* is used for pain relief, chills, flu, fever, muscle ache and skin disorders, asthma, obesity, and diarrhoea [62].

	*Scleria striatinus* (Cyperaceae)	Use against eye infections.

	*Tetrapleura tetraptera* (Fabaceae)	Used to treat epilepsy, convulsions, malaria, fever, and fibromyoma [63].

	*Xylopia aethiopica* (Annonaceae)	Used to treat bronchitis, dysentery, inflammations, cough, and postnatal pains [23].

**Table 2:** Plant extracts or hydrolates and codes: extracts were prepared by air-drying fresh plant material, crushing into powder, and extracting by macerating the powder in hexane, followed by filtration and evaporation. The concentrate was macerated in methanol, filtered, and evaporated and the resulting concentrated crude extracts were coded as shown and stored at 4°C until required for use. Hydrolates were obtained by separating the aqueous water from essential oils after steam distillation in the course of extracting essential oils.

Code	Plant species	Extract or hydrolate
E01	*Xylopia aethiopica *	Methanol fruit extract
E02	*Mitracarpus scaber *	Methanol whole plant extract
E03	*Aframomum danielli *	Methanol seed extract
E04	*Cinnamomum zeylanicum *	Methanol root extract
E05	*Albizia lebbeck *	Methanol bark extract
E06	*Baillonella toxisperma *	Methanol bark extract
E07	*Fagara leprieuri *	Methanol fruit extract
E08	*Kigelia africana *	Methanol stem bark extract
E09	*Tetrapleura tetraptera *	Methanol fruit extract
E10	*Xylopia aethiopica *	Methanol stem bark extract
E11	*Aucoumea klaineana (Okoumé) *	Ethanol resin extract
E12	*Pamplemousse pepin *	Methanol seed extract
E13	*Myrianthus arboreus *	Methanol root extract
E14	*Clausena anisata *	Hexane leaf extract
E15	*Scleria striatinus *	Methanol root extract
E16	*Canarium schweinfurtii *	Gum resin hydrolate
E17	*Aframomum sulcatum *	Seed hydrolate
E18	*Aframomum melegueta *	Seed hydrolate
E19	*Fagara xanthoxyloides *	Fruit hydrolate
E20	*Scleria striatinus *	Hexane root extract
E21	*Piper nigrum *	Fruit hydrolate
E22	*Pentadiplandra brazzeana *	Root hydrolate
E23	*Echinops giganteus *	Root hydrolate
E24	*Cupressus leylandii *	Leaf hydrolate
E25	*Eugenia caryophyllus *	Fruit hydrolate
E26	*Cymbopogon winterianus *	Leaf hydrolate
E27	*Aframomum kayserianum *	Seed hydrolate
E28	*Piper guineense *	Fruit hydrolate
E29	*Piper capense *	Fruit hydrolate
E30	*Nauclea pobeguinii *	Methanol root extract

**Table 3:** Antimicrobial activity of extracts E01–E14:the hole-plate diffusion method was used to assess the antimicrobial susceptibility of crude extracts by measuring the diameter zones of inhibition in mm. The absolute values of the diameter of zones inhibition (DZI) varied from 0 to 25 mm.

Code	*C. albicans *	*S. saprophyticus *	*S. epidermidis *	*S. typhi *	*S. aureus *	*K. pneumonia *	*E. coli *
E01	9	—	—	4	—	—	19
E02	5	7	5	4	7	4	9
E03	6	11	1	4	1	—	25
E04	4	4	2	1	3	—	14
E05	8	8	11	9	8	9	7
E06	14	14	13	9	12	9	9
E07	4	1	—	—	—	—	14
E08	4	9	1	9	6	5	11
E09	3	3	3	2	1	4	4
E10	4	—	—	—	—	—	16
E11	—	1	—	—	—	—	9
E12	9	12	—	—	—	—	12
E13	—	5	4	5	—	1	9
E14	—	1	—	—	—	—	12
+ctl	17	17	14	12	17	25	25
−ctl	—	—	—	—	—	—	—

Values are mean standard deviation of duplicate assays (±1). — = zero zone of inhibition observed. +Ctl = positive control (gentamycin for bacteria and nystatin for *C. albicans*); −Ctl = negative control (50% v/v DMSO).

**Table 4:** Minimal Inhibitory Concentrations (MICs) and Bactericidal Concentrations (MBCs) of crude extracts (*µ*g/mL).

Code	*Trichophyton rubrum *		*Candida albicans *		*S. saprophyticus *		*S. aureus *		*S. epidermidis *		*E. coli *		*Klebsiella pneumoniae *		*Salmonella typhi *	
	MIC	MBC	MIC	MBC	MIC	MBC	MIC	MBC	MIC	MBC	MIC	MBC	MIC	MBC	MIC	MBC
E01	—	—	—	—	3	2	2	1	1	—	3	2	—	—	2	1
E02	4	3	4	3	4	3	3	3	1	—	6	5	1	—	2	1
E03	3	2	3	2	3	2	2	1	1	—	2	1	1	—	2	1
E04	3	2	2	1	3	2	2	1	1	—	4	3	—	—	3	2
E05	6	5	2	1	6	5	4	3	3	2	6	5	3	2	3	2
E06	5	4	3	2	3	2	5	4	2	1	7	6	4	3	3	2
E07	3	2	2	1	6	5	7	6	3	2	4	3	1	—	2	1
E08	3	2	2	1	5	4	3	2	2	1	5	4	2	1	2	1
E09	2	1	1	—	3	2	2	1	1	—	3	2	1	—	2	1
E10	2	1	2	1	4	3	2	1	2	1	6	5	1	—	1	—
E11	—	—	—	—	3	2	1	—	2	1	3	2	—	—	—	—
E12	3	2	4	3	4	3	2	1	2	1	4	3	1	—	3	2
E13	4	3	2	1	5	4	3	2	2	1	6	5	3	2	2	1
E14	1	—	2	1	3	2	2	1	1	—	4	3	1	—	2	1

MICs and MBCs are given to the nearest whole numbers: 1 = 1667 *µ*g/mL; 2 = 556 *µ*g/mL; 3 = 185 *µ*g/mL; 4 = 62 *µ*g/mL; 5 = 21 *µ*g/mL; 6 = 7 *µ*g/mL; 7 = 2 *µ*g/mL. — = no result.

**Table 5:** Minimal Inhibitory Concentrations (MICs) and Minimal Bactericidal Concentrations (MBCs) of liquid extracts and hydrolates (parts per million, ppm).

Code	*Tricophyton rubrum *		*Candida albicans *		*Staphylococcus saprophyticus *		*S. aureus *		*S. epidermidis *		*E. coli *		*Klebsiella pneumoniae *		*Salmonella typhi *	
	MIC	MBC	MIC	MBC	MIC	MBC	MIC	MBC	MIC	MBC	MIC	MBC	MIC	MBC	MIC	MBC
E15	4	3	4	3	7	6	4	3	3	2	7	6	1	—	5	4
E16	—		4	3	2	1	2	1	3	2	—	—	—	—	1	—
E17	5	4	4	3	6	5	6	5	3	2	7	6	4	3	3	2
E18	—		—		2	1	—	—	—	—	3	2	—	—	—	—
E19	—		—		1	—	—	—	—	—	3	2	—	—	4	3
E20	5	4	4	3	6	5	2	1	3	2	5	4	1	—	—	—
E21	—	—	—	—	—	—	—	—	—	—	—	—	—	—	—	—
E22	—	—	—	—	—	—	—	—	—	—	—	—	—	—	—	—
E23	—	—	—	—	—	—	—	—	—	—	—	—	—	—	—	—
E24	—	—	—	—	—	—	—	—	—	—	—	—	—	—	—	—
E25	—	—	—	—	—	—	—	—	—	—	—	—	—	—	—	—
E26	—	—	—	—	—	—	—	—	—	—	—	—	—	—	—	—
E27	—	—	—	—	—	—	—	—	—	—	—	—	—	—	—	—
E28	2	1	6	5	2	1	2	1	1	—	3	2	1	—	5	4
E29	3	3	2	1	6	5	3	2	2	1	7	6	4	3	5	4
E30	7	7	4	3	11	10	5	4	4	3	10	9	4	3	4	3

1 = 333333 ppm, 2 = 111111 ppm, 3 = 37037 ppm, 4 = 12346 ppm, 5 = 4115 ppm, 6 = 1372 ppm, 7 = 457 ppm, 8 = 152 ppm, 9 = 51 ppm, 10 = 17 ppm, and 11 = 6 ppm; — = no result. MICs and MBCs are given to the nearest whole number.

**Table 6:** Diameter zone of inhibition of antibiotic discs against bacteria species and resistance status.

Antibiotic	Diameter zone of inhibition (DZI) in mm					
	Bacterium					
	*S. epidermidis *	*S. aureus *	*S. saprophyticus *	*E. coli *	*K. pneumoniae *	*S. typhi *
Ceftriaxone	22 (R)	25 (I)	38 (S)	25 (S)	24 (S)	20
Amoxicillin	6 (R)	6 (R)	6 (R)	30 (S)	6 (R)	6 (I)
Ampicillin	6 (R)	6 (R)	17 (S)	30 (S)	6 (R)	6 (R)
Norfloxacin	32 (S)	30 (S)	36 (S)	10 (R)	31 (S)	30 (S)

R = resistant; I = intermediate; S = sensitive. *S. epidermidis* resistant to Ceftriaxone (CRO), Amoxicillin (AMX), and Ampicillin (AMP); *S. aureus* resistant to CRO, AMX, and AMP; *S. saprophyticus* resistant to AMX; *E. coli* resistant to Norfloxacin (NOR); *K. pneumonia* resistant to AMX and AMP; *S. typhi* resistant to CRO, AMX, and AMP.

**Table 7:** Minimal Inhibitory Concentrations of resistant antibiotics.

Antibiotic	Bacterium					
	*S. epidermidis *	*S. aureus *	*S. saprophyticus *	*E. coli *	*K. pneumoniae *	*S. typhi *
Ceftriaxone	11	11	ND	ND	ND	7
Amoxicillin	3	4	11	ND	3	2
Ampicillin	3	3	ND	ND	3	3
Norfloxacin	ND	ND	ND	11	ND	ND

2 = 2.2 *μ*g/mL, 3 = 0.74 *μ*g/mL, 4 = 0.25 *μ*g/mL, 7 = 0.0091 *μ*g/mL, 11 = 1.13 × 10^−4^ *μ*g/mL, and ND = not determined.

**Table 8:** Synergism of extracts (E05, E06, E17, and E30) with antibiotics (CRO, AMP, AMX, and NOR) to which bacteria is resistant.

Test organism	Extract	Test	MIC	Result of the combination
*S. aureus *	*Albizia lebbeck* (E05)	E05	1	
		E05 + CRO	1	No effect
		CRO	1	
		E05 + AMP	2	Synergism
		AMP	1	
		E05 + AMX	1	No effect
		AMX	1	
	*Baillonella toxisperma* (E06)	E06	1	
		E06 + CRO	1	No effect
		E06 + AMP	1	No effect
		E06 + AMX	1	No effect
	*Aframomum sulcatum* (E17)	E17	1	
		E17 + CRO	7	Synergism
		E17 + AMP	1	No effect
		E17 + AMX	1	No effect
	*Nauclea pobeguinii* (E30)	E30	1	
		E30 + CRO	11	Synergism
		E30 + AMP	6	Synergism
		E30 + AMX	3	Synergism

*S. saprophyticus *	E05	E05	1	
		E05 + AMX	—	Antagonism
		AMX	1	
	E06	E06	1	
		E06 + AMX	—	Antagonism
	E17	E17	1	
		E17 + AMX	—	Antagonism
	E30	E30	1	
		E30 + AMX	—	Antagonism

*E. coli *	E05	E05	1	
		E05 + NOR	—	Antagonism
		NOR	1	
	E06	E06	1	
		E06 + NOR	—	Antagonism
	E17	E17	1	
		E17 + NOR	2	Synergism
	E30	E30	1	
		E30 + NOR	8	Synergism

*K. pneumoniae *	E05	E05	1	
		E05 + AMP	1	No effect
		AMP	1	
		E05 + AMX	—	Antagonism
		AMX	1	
	E06	EO6	1	
		E06 + AMP	1	No effect
		E06 + AMX	—	Antagonism
	E17	E17	1	
		E17 + AMP	1	No effect
		E17 + AMX	—	Antagonism
	E30	E30	1	
		E30 + AMP	2	Synergism
		E30 + AMX	2	Synergism

*S. epidermidis *	E05	E05	1	
		E05 + CRO	1	No effect
		CRO	1	
		E05 + AMP	1	No effect
		AMP	1	
		E05 + AMX	1	No effect
		AMX	1	
	E06	E06	1	
		E06 + CRO	2	Synergism
		E06 + AMP	2	Synergism
		E06 + AMX	1	No effect
	E17	E17	1	
		E17 + CRO	—	Antagonism
		E17 + AMP	—	Antagonism
		E17 + AMX	1	No effect
	E30	E30	1	
		E30 + CRO	—	Antagonism
		E30 + AMP	3	Synergism
		E30 + AMX	—	Antagonism

*S. typhi *	E05	E05	1	
		E05 + CRO	1	No effect
		CRO	1	
		E05 + AMP	1	No effect
		AMP	1	
		E05 + AMX	1	No effect
		AMX	1	
	E06	E06	1	
		E06 + CRO	1	No effect
		E06 + AMP	—	Antagonism
		E06 + AMX	—	Antagonism
	E17	E17	1	
		E17 + CRO	1	No effect
		E17 + AMP	1	No effect
		E17 + AMX	2	Synergism
	E30	E30	1	
		E30 + CRO	4	Synergism
		E30 + AMP	1	No effect
		E30 + AMX	1	No effect

1 = concentration in well one (MIC of antibiotic alone, extract alone, or extract and antibiotic); 2 = 1/3 MIC; 3 = 1/9 MIC; 4 = 1/27 MIC; 7 = 1/243 MIC; 8 = 1/1944 MIC; 11 = MIC × 3^−10^.

**Table 9:** Chemical composition of active extracts E05, E06, E17, and E30.

Chemical constituent	E05	E06	E17	E30
Alcaloids	+	+	+++	+++
Phenols	+	+++	—	—
Triterpenes	+	++	+++	—
Steroids	—	—	++	+++
Coumarines	—	—	—	—
Flavonoids	+	+++	+	—
Saponines	++	+++	—	—
Glycosides	+	+	+	—

+++: abundant; ++: average; +: in traces; —: absent.
